# Urinary bacterial spectrum and antibiotic resistance trends at a Urology Clinic in Hungary between 2012 and 2023

**DOI:** 10.1007/s11255-025-04683-z

**Published:** 2025-07-25

**Authors:** Ádám Miklós Fehér, Milad Safikhani, Zoltán Bajory, Andrea Lázár, Katalin Burián, Ferenc Rárosi, Béla Köves

**Affiliations:** 1 Department of Urology, University of Szeged, Szeged, Hungary; 2 Department of Medical Microbiology, University of Szeged, Szeged, Hungary; 3 Department of Medical Physics and Informatics, University of Szeged, Szeged, Hungary; 4 Department of Urology, Jahn Ferenc South Pest Teaching Hospital, Budapest, Hungary

**Keywords:** Bacterial spectrum, Antibiotic resistance, Urinary tract infection, Multidrug-resistant bacterium, Surveillance, Antibiotic stewardship

## Abstract

**Purpose:**

Widespread antibiotic use has promoted a concerning rise in bacterial resistance. To counteract this trend and improve the effectiveness of antibiotic treatments, implementing antibiotic stewardship and an active surveillance system is crucial. Our primary aim was to analyze the local urinary bacterial spectrum and antibiotic resistance trends.

**Methods:**

All positive urine culture results (9423) obtained between January 1, 2012 and December 31, 2023 at the Urology Department, University of Szeged were analyzed. Spearman’s rank correlation test was then used to examine changes in bacterial spectrum, resistance trends of the five most common bacteria, and incidences of multidrug-resistant strains and nosocomial *Clostridioides difficile* infections.

**Results:**

The proportion of *Escherichia coli* decreased significantly from 53 to 40% (*p* < 0.001), whereas that of *Proteus mirabilis* increased from 4 to 6% (*p* = 0.018). We observed significant decreases in *E. coli* for amoxicillin/clavulanic acid (*p* = 0.011), cefuroxime (*p* = 0.033), and gentamicin (*p* < 0.001); *Enterococcus faecalis* for gentamicin (*p* = 0.002); *Klebsiella pneumoniae* for amoxicillin/clavulanic acid, cefuroxime, ceftriaxone, ciprofloxacin, gentamicin, and trimethoprim/sulfamethoxazole (*p* < 0.001); *Pseudomonas aeruginosa* for ciprofloxacin (*p* = 0.001) and gentamicin (*p* = 0.006); and *P. mirabilis* for amoxicillin/clavulanic acid (*p* = 0.018) and trimethoprim/sulfamethoxazole (*p* = 0.022). Only *K. pneumoniae* showed a significant increase in resistance trends (fosfomycin, *p* < 0.001). The incidence of extended-spectrum beta-lactamase-producing bacteria decreased significantly from 14.35% to 8.92% (*p* < 0.001), whereas that of *C. difficile* infections decreased by two-thirds.

**Conclusion:**

Antibiotic stewardship based on accurate surveillance can counteract the increasing trend of antibiotic resistance and help manage urinary tract infections in the future, as well as might reduce the incidence of multidrug-resistant strains and *C. difficile*.

## Introduction

The increasing trend in antimicrobial resistance (AMR) has emerged as a worldwide problem that negatively impacts both healthcare systems and economies. Without the development of novel antibiotics in the near future, several patients may not receive effective treatment for multidrug-resistant (MDR) bacterial infections.

Urinary tract infections (UTIs) are among the most common diseases caused by bacterial infections, affecting up to 27% of the healthy adult population. The prevalence UTIs is even higher among elderly and institutionalized individuals (15–50%) who are also more vulnerable to complications [1]. Managing MDR UTIs in hospitals is becoming increasingly challenging, which can prolong the duration of antibiotic treatments, extended hospitalization, raise costs, and increased mortality rates [2]. In the United States alone, the total expenses associated with UTIs, including medications, hospital care, and missing workdays, exceed $5 billion [3].

Therefore, understanding and analyzing the local bacterial spectrum and resistance have become a priority, which allows clinicians to provide more accurate treatments that decrease morbidity and mortality rates and costs. Moreover, effective antibiotic stewardship remains crucial in slowing the emergence of resistant strains. The current study aimed to retrospectively analyze the longitudinal trends in our local urinary culture data and compare them to those observed in other regional results.

## Materials and methods

### Ethical considerations

The single-center, retrospective observational study was conducted in accordance with the Declaration of Helsinki and the principles of Good Clinical Practice and was approved by the local ethics committee (44/2023-SZTE RKEB, approved: 20-Mar-2023).

All positive urine culture results collected between January 1, 2012 and December 31, 2023 from the Urology Department, University of Szeged were analyzed. We analyzed outpatient and inpatient samples, as well as the prevalence of nosocomial *C. difficile* infections. All data were collected from the electronic medical records (MedBakter, Asseco Magyarország) of the Department of Medical Microbiology, University of Szeged. The five most common bacteria strains were evaluated.

### Urine culture method

After obtaining the urine samples at the Department of Urology, boric acid containers were used to transform them to the Department of Medical Microbiology. Inoculation was performed using a 0.01-mL loop onto URISELECT (BioRad) agar media. Samples were incubated at 35 ± 2 °C in normal atmosphere. Bacteria were identified using a matrix-assisted laser desorption ionization time-of-flight mass spectrometry system (MALDI-TOF MS) device (Bruker Daltonics Gmbh) since July 2012. Before July 2012, conventional biochemical tests or the VITEK 2 automated system (Biomerieux) were used for bacterial identification. Antibiotic sensitivity tests were performed using the disc diffusion test method according to the current standards of the European Committee on Antimicrobial Susceptibility Testing (EUCAST) [4].

The following MDR bacteria were detected: extended-spectrum beta-lactamase (ESBL)-producing bacteria, multiresistant *Acinetobacter baumannii* (MACI), multiresistant *Pseudomonas aeruginosa* (MPAE), methicillin-resistant *Staphylococcus aureus* (MRSA), and vancomycin-resistant *Enterococcus* (VRE).

### Statistical analysis

Continuous variables were expressed as mean ± SD, whereas categorical data were expressed as number of cases (frequency) and percentage (relative frequency). Spearman’s rank correlation analysis was used to examine overall tendencies in incidence rates and year and antibiotic resistance rates and year. Sign (tendency) and significance of the Spearman’s rank correlation coefficients were evaluated. All analyses were conducted using IBM SPSS Statistics version 29.0.0.0 (241), with *p* values < 0.05 indicating statistical significance.

## Results

During the 12-year period, 8596 positive urine cultures with 9423 bacterial isolates were collected and evaluated. Among the 827 cases included, more than one bacterium had been detected simultaneously. The annual number of the positive samples generally increased from 2012 to 2019; however, the COVID-19 pandemic caused a significant drop in the number of positive samples until 2023, when the number of positive samples started to increase again.

### Bacterial spectrum

**Fig. 1:**
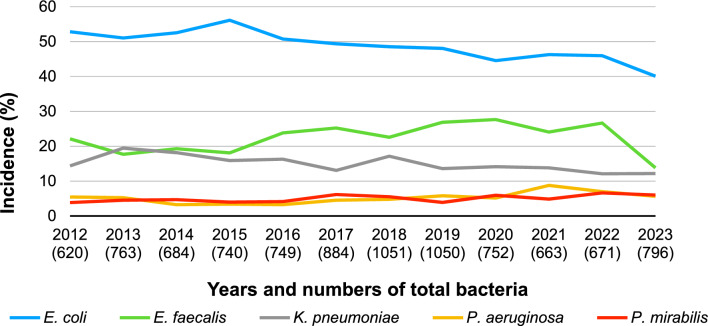
Yearly incidence of the five most common bacteria

The proportion on Gram-negative and -positive bacteria remained relatively stable during the 12-year period (~ 75% vs. ~ 25%, respectively). The five most commonly detected bacterial species (*Escherichia coli*, *Enterococcus faecalis*, *Klebsiella pneumoniae*, *P. aeruginosa*, and *Proteus mirabilis*) were statistically analyzed in terms of their yearly distribution (Fig. 1) and antibiotic resistance.

The most frequently detected bacterium was *E. coli*, with an incidence rate of approximately 50%. However, its proportion significantly declined from 53 to 40% (Spearman’s rho = − 0,930, very strong negative tendency, 95% CI − 0.981, − 0.764, *p* < 0.001). The overall proportions of *E. faecalis* and *K. pneumoniae* also decreased (from 22 to 14% and from 14 to 12%, respectively), but only the latter showed a significant trend (Spearman’s rho = − 0.748, strong negative tendency, 95% CI − 0.925, − 0.305, *p* = 0.005). In contrast, the proportion of *P. aeruginosa* slightly increased (from 5 to 6%, *p* = 0.051), whereas that of *P. mirabilis* significantly increased (from 4 to 6%, Spearman’s rho = 0.664, strong positive tendency, 95% CI 0.146, 0.896, *p* = 0.018). These five bacterial species were detected in more than 95% of the samples throughout the study period, except in 2023 (~ 78%).

**Fig. 2:**
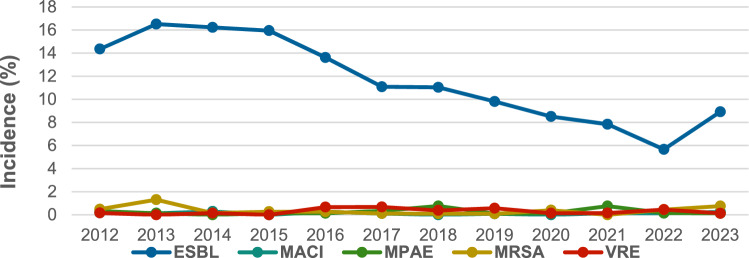
Incidence of MDR bacteria. *ESBL* extended spectrum of beta-lactamase, *MACI* multiresistant *Acinetobacter baumannii*, *MPAE* multiresistant *Pseudomonas aeruginosa*, *MRSA* methicillin-resistant *Staphylococcus aureus*, *VRE* vancomycin-resistant *Enterococcus*

Among MDR bacteria, the ESBL-producing strains decreased significantly over the 12-year period, declining from 14.35% to 8.92% by 2023 (Spearman’s rho = − 0,916, very strong negative tendency, 95% CI − 0.977, − 0.721, *p* < 0.001). MRSA exhibited a single spike in detection in 2013 (10/763 bacteria, 1.31%); otherwise, its presence remained between 0% and 0.75% (Fig. 2).

Moreover, nosocomial *C. difficile* infections were also observed, with the annual number decreasing from 17 to 6 cases between 2013 and 2023.

### Antibiotic resistance trends

Clinically relevant antibiotics, such as amoxicillin/clavulanic acid (AMC), cefuroxime, ceftriaxone, cefepime, ciprofloxacin, gentamicin, imipenem, ertapenem, meropenem, trimethoprim/sulfamethoxazole (TMP/SMX), fosfomycin, and nitrofurantoin, and their effectiveness were evaluated according to the current EUCAST breakpoints. The “intermediately susceptible” category was reported as susceptible in our results [4]. Detailed annual data and trends are shown in Tables 1, 2, 3, 4, and 5. We only highlight significant changes or important data informing antibiotic policy in the text.

### *E. coli*

Our results showed a decline in all examined antibiotic resistance rates, except for fosfomycin (Table 1). Resistance to AMC and cefuroxime decreased significantly from 24.67% to 18.18% (Spearman’s rho = − 0.699, strong negative tendency, 95% CI −0.908; − 0.209, *p* = 0.011) and from 15.61% to 13.21% (Spearman’s rho = − 0.615, strong negative tendency, 95% CI − 0.879, − 0.064, *p* = 0.03, *p* = 0.033), respectively. Gentamicin resistance decreased from 12% to 5.64% (Spearman’s rho = − 0.853, very strong negative tendency, 95% CI − 0.958, − 0.547, *p* < 0.001). Nitrofurantoin and fosfomycin resistance rates remained generally low (2.44% and 3.15%, in 2023, respectively), although the latter was rarely tested. Among the carbapenems, ertapenem was primarily tested, which showed a resistance rate of 0%.

**Table 1:** Yearly resistance rates of *E. coli* (*EC*)

EC	AMC			CFX			CTX			CIP			GM			ERT			TMP/SMX			FM			NF		
	%	n	N	%	n	N	%	n	N	%	n	N	%	n	N	%	n	N	%	n	N	%	n	N	%	n	N
2012	24.67	74	300	15.61	47	301	13.95	42	301	33.33	100	300	12.00	36	300	0	0	299	28.67	82	286	0	0	3	6.40	19	297
2013	21.49	75	349	14.61	51	349	12.93	45	348	36.68	128	349	10.95	38	347	0	0	347	34.05	111	326	7.14	2	28	4.35	15	345
2014	19.81	64	323	15.48	50	323	14.81	48	324	38.27	124	324	12.46	40	321	0	0	324	29.19	94	322	0	0	18	5.38	17	316
2015	18.42	70	380	15.49	59	381	14.44	55	381	40.42	154	381	12,07	46	381	0	0	381	37.40	141	377	4,17	1	24	3.45	13	377
2016	18.95	65	343	16.03	55	343	15.45	53	343	41.11	141	343	11.37	39	343	0	0	343	39.65	136	343	2.78	1	36	1.18	4	339
2017	17.30	68	393	13.99	55	393	12.98	51	393	49.23	193	392	6.87	27	393	0	0	393	35.64	139	390	0	0	45	0.52	2	388
2018	19.10	85	445	15.28	68	445	14.38	64	445	40.22	179	445	7.21	32	444	0	0	445	33.03	145	439	0	0	57	1.59	7	439
2019	21.58	93	431	17.87	77	431	16.24	70	431	40.93	176	430	9.05	39	431	0	0	431	37.21	160	430	4.41	3	68	1.41	6	425
2020	15.16	42	277	12.41	34	274	11.91	33	277	31.05	86	277	8.00	22	275	0	0	277	32.25	89	276	3.33	1	30	2.95	8	271
2021	13.31	33	248	11.29	28	248	10.08	25	248	25.81	64	248	6.48	16	247	0	0	248	40.73	101	248	0	0	28	2.85	7	246
2022	16.87	41	243	11.52	28	243	11.11	27	243	24.69	60	243	4.94	12	243	0	0	243	30.17	73	242	0	0	30	2.52	6	238
2023	18.18	58	319	13.21	42	318	12.54	40	319	22.57	72	319	5.64	18	319	0	0	319	26.18	83	317	2.44	1	41	3.15	10	312
Δ, rho, *p*	− 6.49	− 0.699	**0.011**	− 2.40	− 0.615	**0.033**	− 1.41	− 0.517	0.085	− 10.76	− 0.490	0.106	− 6.36	− 0.853	**< 0.001**	0	–	–	− 2.49	− 0.028	0.931	2.44	− 0.127	0.694	− 3.25	− 0.448	0.145

*AMC* amoxicillin/clavulanic acid, *CFX* cefuroxime, *CTX* ceftriaxone, *CIP* ciprofloxacin, *GM* gentamicin, *ERT* ertapenem, *TMP/SMX* trimethoprim/sulfamethoxazole, *FM* fosfomycin, *NF* nitrofurantoin, *Δ* difference between 2012 and 2023, *rho* Spearman’s correlation coefficient, *p* trend significance

### *E. faecalis*

*E. faecalis* exhibits intrinsic susceptibility to aminopenicillin derivatives and resistance to cephalosporins. Resistance rates to high-dose gentamicin significantly decreased from approximately 45%–50% to 30% (Spearman’s rho = − 0.790, strong negative tendency, 95% CI − 0.938, 0.395, *p* = 0.002). Owing to its high resistance rates, TMP/SMX was tested only until 2014, according to EUCAST guidance (Table 2).

**Table 2:** Yearly resistance rates of *E. faecalis* (*EF*)

EF	CIP			GM			TMP/SMX			NF		
	%	n	N	%	n	N	%	n	N	%	n	N
2012	45.45	5	11	44.00	55	125	41.77	33	79	0	0	119
2013	N.d	N.d	N.d	49.59	60	121	42.98	49	114	0	0	106
2014	20.99	17	81	59.66	71	119	0	0	9	0.91	1	110
2015	30.00	30	100	53.66	66	123	N.d	N.d	N.d	0.82	1	122
2016	24.82	34	137	48.13	77	160	N.d	N.d	N.d	1.31	2	153
2017	48.70	94	193	48.26	97	201	100	1	1	0.51	1	197
2018	38.65	80	207	46.63	97	208	N.d	N.d	N.d	0.98	2	205
2019	39.58	95	240	42.50	102	240	N.d	N.d	N.d	1.28	3	235
2020	30.12	50	166	29.65	51	172	N.d	N.d	N.d	0	0	147
2021	32.81	42	128	34.11	44	129	N.d	N.d	N.d	0	0	126
2022	18.38	25	136	28.06	39	139	N.d	N.d	N.d	0.78	1	129
2023	20.18	22	109	30.00	33	110	N.d	N.d	N.d	0	0	103
Δ, rho, *p*	− 25.27	− 0.364	0.272	14.00	− 0.790	**0.002**	–	–	–	0	− 0.109	0.737

*CIP* ciprofloxacin, *GM* gentamicin, *TMP/SMX* trimethoprim/sulfamethoxazole, *NF* nitrofurantoin, *Δ* difference between 2012 and 2023, *rho* Spearman’s correlation coefficient, *p* trend significance, *n.d.* no data

### *K. pneumoniae*

Baseline resistance rates of *K. pneumoniae* were high, exceeding 60% for most antibiotics, except for fosfomycin and ertapenem. However, significant decreases were observed for AMC (Spearman’s rho = − 0.909, very strong negative tendency, 95% CI − 0.975, − 0.701, *p* < 0.001), cefuroxime (Spearman’s rho = − 0.944, very strong negative tendency, 95% CI − 0.985, −0.808, *p* < 0.001), ceftriaxone (Spearman’s rho = −0.909, very strong negative tendency, 95% CI: −0.975, −0.701, *p* < 0.001), ciprofloxacin (Spearman’s rho = − 0.923, very strong negative tendency, 95% CI: −0.979, −0.742, *p* < 0.001), gentamicin (Spearman’s rho = −0.965, very strong negative tendency, 95% CI − 0.990, − 0.877, *p* < 0.001), and TMP/SMX (Spearman’s rho = − 0.895, very strong negative tendency, 95% CI − 0.970, − 0.660, *p* < 0.001). Fosfomycin resistance rates increased significantly (Spearman’s rho = 0.879, very strong positive tendency, 95% CI 0.616, 0.966, *p* < 0.001), whereas ertapenem resistance rates remained below 2% (Table 3).

**Table 3:** Yearly resistance rates of *K. pneumoniae* (*KP*)

*KP*	ACM			CFX			CTX			CP			CIP			GM			ERT			TMP/SMX			FM		
	%	n	N	%	n	N	%	n	N	%	n	N	%	n	N	%	n	N	%	n	N	%	n	N	%	n	N
2012	67.90	55	81	68.29	56	82	65.85	54	82	76.92	10	13	71.95	59	82	62.34	48	77	0	0	82	70.13	54	78	14.29	2	14
2013	65.41	87	133	66.67	88	132	65.41	87	133	100	9	9	69.92	93	133	53.54	68	127	1.54	2	130	62.50	75	120	20.00	13	65
2014	68.75	77	112	66.07	74	112	66.07	74	112	N.d	N.d	N.d	71.43	80	112	52.73	58	110	0.90	1	111	66.67	74	111	40.35	23	57
2015	67.59	73	108	65.42	70	107	65.74	71	108	100	2	2	73.15	79	108	51.85	56	108	0	0	108	63.89	69	108	40.00	20	50
2016	59.09	65	110	56.36	62	110	54.55	60	110	75.00	3	4	62.73	69	110	44.55	49	110	0	0	110	63.30	69	109	40.00	20	50
2017	51.92	54	104	45.19	47	104	45.19	47	104	100	5	5	62.50	65	104	48.08	50	104	N.d	N.d	N.d	58.65	61	104	58.33	14	24
2018	37.34	59	158	35.44	56	158	35.44	56	158	50	2	4	46.20	73	158	33.54	53	158	0	0	158	49.68	78	157	43.59	17	39
2019	31.97	39	122	31.15	38	122	31.15	38	122	50	1	2	39.34	48	122	18.03	22	122	0.82	1	122	27.87	34	122	53.13	17	32
2020	35.23	31	88	35.23	31	88	34.09	30	88	0	0	1	32.95	29	88	27.27	24	88	0	0	88	40.91	36	88	50.00	12	24
2021	40.54	30	74	40.54	30	74	39.19	29	74	0	0	1	40.54	30	74	24.32	18	74	1.35	1	74	44.59	33	74	46.67	14	30
2022	17.19	11	64	17.46	11	63	15.63	10	64	0	0	1	22.22	14	63	9.38	6	64	1.56	1	64	23.44	15	64	60.00	9	15
2023	30.93	30	97	28.87	28	97	27.84	27	97	N.d	N.d	N.d	29.90	29	97	14.43	14	97	1.03	1	97	37.89	36	95	75.00	21	28
Δ, rho, *p*	− 36.97	− 0.909	**< 0.001**	− 39.42	− 0.944	**< 0.001**	− 38.01	− 0.909	**< 0.001**	–	–	–	− 42.05	− 0.923	**< 0.001**	− 47.91	− 0.965	**< 0.001**	1.03	0.362	0.276	− 32.24	− 0.895	**< 0.001**	60.71	0.879	**< 0.001**

*AMC* amoxicillin/clavulanic acid, *CFX* cefuroxime, *CTX* ceftriaxone, *CP* cefepime, *CIP* ciprofloxacin, *GM* gentamicin, *ERT* ertapenem, *TMP/SMX* trimethoprim/sulfamethoxazole, *FM* fosfomycin, *Δ* difference between 2012 and 2023, *rho* Spearman’s correlation coefficient, *p* = trend significance, *n.d.* no data

### *P. aeruginosa*

Resistance to AMC, cefuroxime, ceftriaxone, ertapenem, TMP/SMX, nitrofurantoin, and fosfomycin was not evaluated due to the intrinsic resistance of *P. aeruginosa* to such drugs. Ciprofloxacin resistance rates showed the most substantial decrease across the study period (50.18%), with the decreased being highly significant (Spearman’s rho = − 0.839, very strong negative tendency, 95% CI − 0.954, − 0.511, p = 0.001). Gentamicin only had EUCAST guidance until 2020, although it showed a significant 46.52% decrease (Spearman’s rho = − 0.794, strong negative tendency, 95% CI − 0.949, − 0.329, *p* = 0.006). Imipenem and meropenem had similar resistance rates, varying irregularly across a wide range (0–34.09%) (Table 4).

**Table 4:** Yearly resistance rates of *P. aeruginosa* (*PA*)

*PA*	CP			CIP			GM*			IMI			MER		
	%	n	N	%	n	N	%	n	N	%	n	N	%	n	N
2012	29.03	9	31	61.29	19	31	58.06	18	31	22.58	7	31	25.81	8	31
2013	0	0	36	61.11	22	36	38.89	14	36	13.89	5	36	8.33	3	36
2014	0	0	20	65.00	13	20	45.00	9	20	15.00	3	20	0	0	20
2015	4.35	1	23	56.52	13	23	52.17	12	23	21.74	5	23	8.70	2	23
2016	18.18	4	22	68.18	15	22	45.45	10	22	13.64	3	22	18.18	4	22
2017	8.33	3	36	16.67	6	36	13.89	5	36	11.11	4	36	11.11	4	36
2018	25.00	11	44	47.73	21	44	34.09	15	44	34.09	15	44	29.55	13	44
2019	3.85	2	52	32.69	17	52	11.54	6	52	5.77	3	52	7.69	4	52
2020	9.38	3	32	15.63	5	32	0	0	9	3.13	1	32	3.13	1	32
2021	14.89	7	47	29.79	14	47	33.33	1	3	27.66	13	47	29.79	14	47
2022	5.41	2	37	16.22	6	37	N.d	N.d	N.d	5.41	2	37	2.70	1	37
2023	6.67	3	45	11.11	5	45	N.d	N.d	N.d	17.78	8	45	15.56	7	45
Δ, rho, *p*	− 22.36	0.074	0.820	− 50.18	− 0.839	** < 0.001**	− 46.52	− 0.749	**0.006**	− 4.80	− 0.238	0.457	− 10.25	0.007	0.983

*CP* cefepime, *CIP* ciprofloxacin, *GM* gentamicin, *IMI* imipenem, *MER* meropenem, *Δ* difference between 2012 and 2023, *rho* Spearman’s correlation coefficient, *p* trend significance, *n.d.* no data

*Partial results

### *P. mirabilis*

The resistance rates to AMC, cefuroxime, and ceftriaxone followed a similar pattern such that a slight initial increase followed by a decrease until 2022 was noted. In 2023, resistance rates tended to increase, although a significant overall increase was only observed for AMC (Spearman’s rho = − 0.666, strong negative tendency, 95% CI − 0.897, − 0.149, *p* = 0.018). Ciprofloxacin resistance did not significantly change (*p* = 0.145) and remained high at around 50%. Ertapenem resistance was detected in only one strain. TMP/SMX resistance rates consistently remained above 50%, although a significant decrease was observed (Spearman’s rho = − 0.650, strong negative tendency, 95% CI − 0.891, − 0.121, *p* = 0.022). Fosfomycin resistance rates remained high throughout the study, although testing was only performed on a subset of isolates (Table 5).

**Table 5:** Yearly resistance rates of *P. mirabilis* (*PM*)

*PM*	ACM			CFX			CTX			CIP			GM			ERT			TMP/SMX			FM		
	%	n	N	%	n	N	%	n	N	%	n	N	%	n	N	%	n	N	%	n	N	%	n	N
2012	45.45	10	22	45.45	10	22	45.45	10	22	50	11	22	27.27	6	22	0	0	22	90	18	20	33.33	1	3
2013	41.94	13	31	35.48	11	31	35.48	11	31	45.16	14	31	6.45	2	31	0	0	31	60.71	17	28	20.00	1	5
2014	55.17	16	29	51.72	15	29	51.72	15	29	55.17	16	29	37.93	11	29	0	0	29	79.31	23	29	0	0	6
2015	66.67	18	27	66.67	18	27	62.96	17	27	70.37	19	27	18.52	5	27	0	0	27	70.37	19	27	63.64	7	11
2016	39.29	11	28	35.71	10	28	32.14	9	28	46.43	13	28	21.43	6	28	0	0	28	89.29	25	28	50.00	2	4
2017	57.14	28	49	55.10	27	49	51.02	25	49	61.22	30	49	20.83	10	48	0	0	49	81.25	39	48	35.71	5	14
2018	43.14	22	51	44.00	22	50	43.14	22	51	60.78	31	51	13.73	7	51	1.96	1	50	62.75	32	51	50.00	7	14
2019	40.00	14	35	40.00	14	35	31.43	11	35	37.14	13	35	22.86	8	35	0	0	35	57.14	20	35	54.55	6	11
2020	37.84	14	37	37.84	14	37	37.84	14	37	37.84	14	37	29.73	11	37	0	0	37	62.16	23	37	66.67	8	12
2021	26.92	7	26	26.92	7	26	26.92	7	26	42.31	11	26	15.38	4	26	0	0	26	69.23	18	26	71.43	5	7
2022	28.57	10	35	28.57	10	35	28.57	10	35	42.86	15	35	34.29	12	35	0	0	35	54.29	19	35	33.33	3	9
2023	40.00	18	45	38.30	18	47	37.50	18	48	47.92	23	48	16.67	8	48	0	0	48	58.33	28	48	61.11	11	18
Δ, rho, p	− 5.45	− 0.666	**0.018**	− 7.15	− 0.469	0.124	− 7.95	− 0.559	0.059	− 2.08	− 0.448	0.145	− 10.60	− 0.014	0.966	0	0.044	0.893	− 31.67	− 0.650	**0.022**	27.78	0.568	0.054

*AMC* amoxicillin/clavulanic acid, *CFX* cefuroxime, *CTX* ceftriaxone, *CIP* ciprofloxacin, *GM* gentamicin, *ERT* ertapenem, *TMP/SMX* trimethoprim/sulfamethoxazole, *FM* fosfomycin, *Δ* difference between 2012 and 2023, *rho* Spearman’s correlation coefficient, *p* trend significance

## Discussion

Antibiotic resistance is an emerging problem worldwide, highlighting the importance of identifying local bacterial spectrum and antibiotic resistance. To ensure appropriate and cost-effective antimicrobial treatment, strict antibiotic stewardship and surveillance must be implemented locally. Regarding Hungarian studies, Gajdács et al. evaluated *E. coli*, *Klebsiella* species [5], and Gram-positive cocci [3] in UTIs between 2008 and 2017, while the most similar single-center analysis of urinary bacterial flora and AMR rates (2004–2015) was performed by Magyar et al. [6]. The following comparison with our results was limited to the years that coincided with the timeframe of these investigations.

Our results showed a significant decrease in the prevalence of *E. coli* and *K. pneumoniae* but a significant increase in that of while *P. mirabilis*. The observed bacterial spectrum was similar to previously published regional results, which showed that *E. coli* was the most prominent strain followed by *E. faecalis*, *K. pneumoniae*, *P. aeruginosa*, and *P. mirabilis* [5–9].

Among the MDR species, the prevalence of ESBL bacteria decreased significantly, which was contrary to a similar study by Magyar et al. [6]. Gajdács et al. found a pronounced elevation in the prevalence of MRSA in the second half of the study period, which was not consistent with in our results [3]. Evidence has shown that in almost every second of hospital admission, inappropriate antibiotics have been selected, leading to higher costs, longer hospitalization, and increased mortality rates and incidence of nosocomial infections, such as *C. difficile* [1]. However, our findings showed a decrease in incidence rates of *C. difficile*. These results may be attributed to our revised local antibiotic policy introduced around 2016, which mostly replaced the previously used prolonged fluoroquinolone-based preoperative antibiotic prophylaxis with single-shot cephalosporins. Contrary to this, some procedures (e.g., prostate biopsy) have been performed under fluoroquinolone prophylaxis even later. In accordance with this, our study showed a decrease in fluoroquinolone and cephalosporin resistance trends compared to others [5,6], meanwhile fluoroquinolone resistance rates in our country have generally been high (26% for *E. coli* and 38% for *K. pneumoniae*) in 2023 [10].

Resistance rates of *E. coli* to AMC decreased significantly, while others found varying AMR rates (14–35%) [6]. We found relatively low cephalosporin resistance rates (~ 15%), nonetheless significant decrease was only observed in cefuroxime. Ceftriaxone AMR rates were lower in other studies [5,6], except for inpatients [5]. Despite this, our ceftriaxone resistance rate was still superior to the national average in 2023 (12.54% vs. 21.3%) [10]. We found the lowest resistance rates (~ 5%) to gentamicin with a significant decreasing trend among the *E. coli* strains, similarly to the other studies [5,6], except for inpatients, where up to ~ 20% AMR was seen [5]. Ertapenem was extremely efficient against *E. coli* similarly to others [5,6]. *E. coli* showed high susceptibility to nitrofurantoin, which aligns with previous findings [5,6], even in ESBL or AmpC beta-lactamase producers [11]. The low resistance rate to fosfomycin has been supported by a local randomized controlled trial, which administered empiric fosfomycin prophylaxis before transrectal prostate biopsy, with no *E. coli* infections having developed at all after the surgery [12].

AMC is mostly limited to Gram-positive infections, with the most prominent *E. faecalis* having excellent susceptibility to aminopenicillin [3, 6]. Ciprofloxacin resistance rates were similar or higher [3, 6], except for inpatients in Gajdács et al. study [3]. *E. faecalis* showed a significant decrease in resistance rates to gentamicin and showed low AMR rates to nitrofurantoin, which aligns with others’ results [6].

*K. pneumoniae* showed significant decrease in resistance trend of AMC; however, the overall AMR rate was high, similarly to another study [6]. Resistance rates of *K. pneumoniae* to cefuroxime and ceftriaxone decreased significantly. In 2023, AMR rates of *K. pneumoniae* reached > 25% again; however, resistance rates to ceftriaxone were still better than the national average (27.84% vs. 42.0%) [10]. The incidence of fluoroquinolone-resistant *K. pneumoniae* decreased significantly during the study period (from 71.95% to 29.90%); however, more favorable data were reported by others [5,6]. Besides the significant decrease, we found high TMP/SMX resistance rate during the study period, which was similar to that observed in another study [5]. Magyar et al. published fluctuating results [6]. Our findings showed a significant increase in resistance rates of *K. pneumoniae* to fosfomycin. Moreover, our AMR rates to fosfomycin in the *Klebsiella* strains were much higher than in the studies by other authors. [5,6].

The incidence of fluoroquinolone-resistant *P. aeruginosa* decreased pronouncedly over the study period, resulting 11.11% AMR rate in 2013, concurrently the national resistance rate was mostly < 25% [10]. Compared to our results, Magyar et al. found much lower AMR rates to ciprofloxacin and gentamicin [6]. *P. aeruginosa* had moderate but fluctuating resistance to cefepime, meropenem, and imipenem, similarly to the findings of Magyar et al. [6].

The resistance trend of *P. mirabilis* to AMC decreased significantly, although the annual resistance rates remained high especially compared to another study [6]. Magyar et al. found 0% ceftriaxone AMR rate, while our data showed ~ 35–63% during the overlapping years. Although resistance rates of *P. mirabilis* decreased significantly, we found higher than 20% AMR rate similarly to Gajdács et al.[5]. However, *P. mirabilis* showed excellent susceptibility to ertapenem, equally to the results of Magyar et al. [6].

Our findings align with the recommendations of the EAU Guidelines [1]. Empiric use of aminopenicillin is not suggested due to the high AMR rates or negative ecological impact; however, combining with beta-lactamase inhibitors, the preoperative prophylactic use continues to be considered a feasible alternative. Nitrofurantoin and fosfomycin remain useful options for the empiric treatment of uncomplicated lower UTIs, although increased AMR rates, especially in *K. pneumoniae* strains, raise concern. As such, TMP/SMX should only be used when the local resistance rate is below 20%. Except the decreased ciprofloxacin resistance rates observed by our study, the overall AMR rates were high. Moreover, fluoroquinolones are mainly restricted to outpatients with uncomplicated pyelonephritis and prostatitis due to their unfavorable side-effect profile [13]. Cephalosporins seem to be suitable for empiric preoperative prophylaxis, especially considering that our AMR rates have decreased even with their continued use. However, severe UTI or urosepsis requires broad-spectrum empiric antibiotics (e.g., cefepime, AMC + gentamicin, or carbapenems) [1].

## Conclusion

We found mostly decreasing AMR rates during the study period. The only significantly increasing trend was observed in case of *K. pneumoniae* strains to fosfomycin, which should be carefully considered by clinicians. Our results have also shown a decrease in the incidence of MDR bacteria and *C. difficile*. Resistance patterns could be different even in a small country such as Hungary; thus, the knowledge of the local urinary bacterial flora and AMR rates must be crucial. Overall, this research underscores the importance of appropriate antibiotic stewardship, ongoing surveillance, and adaptability in the ongoing fight against antibiotic resistance, which might influence our opportunities in the near future.

## Limitations

Given the changes in the EUCAST breakpoints tables, resistance rates of some strains were no longer evaluated after a certain period. In addition, resistance rates of some strains were less likely tested due to clinical irrelevancy or financial reasons. Occasionally, more than one sample was obtained from a patient at the same time (e.g., different locations: catheter and nephrostomy). In these cases, regarding the five most common species, only in a few samples (0.84%) were identical strains detected, which probably has no significant impact on the results. Our antibiotic policy shifting was implemented as a long process around 2016. For this reason, specific date or year cannot be assigned between our previous and modified antibiotic stewardship. The retrospective design and the longer antibiotic policy shifting process may affect the causality inference of the favorable AMR rates. During the COVID-19 pandemic (2020–2021), a reduction in the number of urine cultures and elective hospital admissions was observed, which could have affected our results.

## Data Availability

The datasets used and analyzed during the current study are available from the corresponding author on reasonable request.
